# Ethical Aspects in Clinical Practice: Recommended Summary Plan for Emergency Care and Treatment (ReSPECT) Conversations and Death Certification Processes Among NHS Clinicians

**DOI:** 10.7759/cureus.101616

**Published:** 2026-01-15

**Authors:** Utsav Balar, Rutva Chauhan, Riya Nilesh Patel

**Affiliations:** 1 Endocrinology, Ashford and St. Peter's Hospitals NHS Foundation Trust, Chertsey, GBR; 2 Acute Medicine, Derriford Hospital, Plymouth, GBR; 3 Medicine, Ashford and St. Peter's Hospitals NHS Foundation Trust, Chertsey, GBR

**Keywords:** doctor-patient communication, emergency care, recommended summary plan for emergency care and treatment (respect), training, treatment planning

## Abstract

Background: The Recommended Summary Plan for Emergency Care and Treatment (ReSPECT) supports shared decision-making about emergency and end-of-life care, while death verification and certification are essential legal and professional responsibilities for clinicians; however, many clinicians feel underprepared for these tasks.

Aim: This study aimed to identify gaps in confidence and training and to contribute to a more thoughtful and compassionate approach in preparing clinicians for some of the most challenging but deeply meaningful parts of their role.

Methods: A cross-sectional online questionnaire-based survey was conducted amongst 109 medical doctors in active clinical roles from across the NHS trusts in the UK. A Cronbach’s alpha value of 0.84 indicated that the questionnaire is a reliable tool for measuring the intended constructs. The data were analysed using IBM SPSS Statistics Version 30.0 (IBM Corp., Armonk, USA).

Results: There were 63 (57.8%) international medical graduates (IMGs) and 46 (42.2%) UK graduates. Time constraint (48.6%) and fear of causing distress (42.2%) were the most common challenges faced during ReSPECT discussions. The majority of the participants had not received any formal training in conducting ReSPECT discussions (74.3%) and death certification processes (60.6%). Workshops/seminars (56.0%) were reported to be the most effective type of training/support pertaining to death verification and certificate forms.

Conclusion: A large proportion of clinicians did not receive proper training in the domains of ReSPECT and death verification. Therefore, greater emphasis should be placed on providing clinicians with appropriate formal training, clear guidelines and protocol, and ongoing support to help them feel more confident in initiating end-of-life discussions.

## Introduction

Medical professionals not only deliver treatment and physical care to relieve suffering but also hold a crucial responsibility in fostering compassionate and continuous dialogue with patients. Through genuine listening and empathetic communication, caregivers can better understand the emotional, social, and existential struggles patients endure, particularly during end-of-life care [1].

Patients nearing the end of life often encounter multifaceted challenges encompassing physical pain, emotional distress, social withdrawal, and existential anxiety. Among the various strategies available, open and honest end-of-life discussions stand out as one of the most significant yet also the most challenging components of compassionate care [1,2]. Existing interventions to support such discussions include national guidance, written protocols, and e-learning resources provided by professional bodies; however, these are often supplemented by informal, experiential learning in clinical practice. A previous study has demonstrated that discussions between physicians and patients about end-of-life decisions positively influence treatment choices, enhance the quality of life for both patients and their families, and reduce the risk of psychiatric disorders among bereaved relatives. Moreover, engaging in such conversations does not heighten the likelihood of patients experiencing depression or anxiety [3].

The Recommended Summary Plan for Emergency Care and Treatment (ReSPECT), introduced by the Resuscitation Council UK (RCUK) in 2016 [4], aims to ensure that emergency care decisions, particularly around resuscitation and treatment escalation, are made collaboratively, respectfully, and in line with a person’s values [5]. It represents a shift from traditional “Do Not Attempt Resuscitation” (DNAR) orders to a more holistic, patient-centered approach. When implemented well, ReSPECT has the potential to enhance clarity, reduce distress, and honor patient autonomy at a time when communication is often most fragile [6]. The approach is grounded in the principles of patient autonomy, person-centered care, and acting in the patient’s best interests [7,8].

Closely linked to this is the clinician’s responsibility for verifying and certifying death [2]. Certifying death not only provides the cause of death but also supports legal, administrative, and epidemiological needs. It is required for estate settlement, insurance claims, and funeral arrangements, and its data helps track disease trends and guide public health policies. Hence, accurate and timely completion is essential. Death certification is legal in nature but deeply human in its implications [9]. These duties are not only procedural; they often mark the final act of care provided to a patient and their family. How this is done can have lasting effects, influencing how families process their loss and how clinicians reflect on their role in that patient’s final journey [2]. Thus, effective end-of-life care communication and accurate death certification are essential clinical responsibilities. Yet, despite their importance, both the ReSPECT process and death documentation are areas where clinicians frequently report feeling under-prepared, under-supported, and emotionally vulnerable [10,11].

A recurring theme from clinicians is the feeling that training has not adequately prepared them for these real-world challenges. Many report learning “on the job” or by observing others, often in moments of crisis or under pressure. While written guidelines and e-learning modules exist, they are rarely experienced as sufficient. Instead, clinicians express a strong preference for interactive, practice-based learning such as simulations, role-playing, observing experienced colleagues in action, and having safe spaces to reflect and debrief [12].

This study explores these issues through the lens of those most affected: the clinicians. The objective of this study was to assess clinicians' confidence, experiences, and training related to the ReSPECT process and death verification within the NHS. By surveying a broad group of doctors working across NHS settings, both UK-trained and internationally educated, we aim to understand the practical and emotional realities they face. 

## Materials and methods

The study was conducted in accordance with institutional governance guidance as an anonymous, questionnaire-based survey of NHS clinicians, involving no patient participation, intervention, or identifiable data.

Study design, study duration, study setting 

This cross-sectional online questionnaire-based survey was conducted between June and August 2025. 

Study population, sample size, sampling design 

This study was conducted amongst medical doctors in active clinical roles from across the NHS trusts in the UK. This study included a sample of 109 clinicians. The sample size was estimated using the following formula: 

$N = \frac{Z_{1-\alpha/2}^2 \times P(1-P)}{d^2}$

where Z = statistic corresponding to the level of confidence; P = prevalence/frequency of occurrence of disease or outcome. For this study, P was taken as 30.0% (percentage of participants confident of initiating a ReSPECT) based on the findings of the pilot study; D = margin of error (considered to be 10%).

Sample size = 1.96 × 1.96 × 0.3 (1 − 0.3) / (0.1)^2^ = 80.6 ⁓81. The minimum required sample size was 81. 

Convenience sampling was used to recruit participants from 10 NHS trusts in the UK, selected based on accessibility and existing professional networks of the research team. A total of 109 clinicians participated, exceeding the minimum required sample size of 81 calculated a priori. Although all trusts contributed respondents, participation varied by site, reflecting natural differences in engagement and survey dissemination.

Questionnaire development 

The information regarding various aspects related to ReSPECT and death certification, including challenges faced by the clinicians in implementing ReSPECT and death verification, confidence in having these discussions, the type of training/support expected, etc., was collected with the help of a questionnaire. The questionnaire was developed in English and primarily consisted of closed-ended items, with a few questions allowing respondents to select multiple options (see Appendices). Comfort and confidence levels were measured using a five-point Likert scale [13], where they were asked to score their comfort and confidence on a scale of 1 to 5. The Likert scale, originally described by Rensis Likert [14], is a widely used public-domain psychometric method for attitude measurement and is freely available for research use without any licensing restrictions. In total, the questionnaire included 21 questions, with the first three designed to capture general demographic information, including educational qualifications, professional role, and years of experience.

The questionnaire was developed with input from subject matter experts to ensure its content validity and relevance to the study objectives. The initial questionnaire draft underwent expert review to ensure content validity, clarity, and methodological suitability. Feedback was obtained from a clinician within one of the participating NHS trust hospitals who has over a decade of research experience, including expertise in questionnaire development and survey methodology. This expert was not a participant in the study and was not involved in data collection. The expert evaluated the questionnaire for clarity of wording, appropriateness of item structure, alignment with the study objectives, and completeness of thematic coverage. Based on this feedback, several refinements were made, including rephrasing ambiguous items, improving the flow of Likert-scale questions, and ensuring comprehensive coverage of key components related to both the ReSPECT process and death verification. This expert input enhanced the overall validity and usability of the final instrument.

After development, the tool was conveniently pilot tested amongst 30 junior doctors, and their responses (to the questions) as well as their recommendations (on how the questions were framed) were used to improve the tool. To assess the internal consistency of the instrument, Cronbach’s alpha was calculated, yielding an acceptable value (0.84), which indicated that the questionnaire was a reliable tool for measuring the intended constructs. 

Data collection 

The self-administered questionnaire was sent to doctors via a link to an online form (Google Forms; Google, LLC, Mountain View, USA) between June to August 2025. The data collection tool requested written informed consent before participants started filling in their responses. No incentives were provided to the participants. The inclusion criteria were medical doctors (foundation year 1 (FY1) and above) who, at the time of data collection, worked in clinical roles and were involved in patient care where ReSPECT discussions or death verification processes may arise. Those doctors who were not currently in clinical practice (e.g., on research posts, career breaks, or non-clinical roles) and those who were not medically qualified, such as nurses, allied healthcare professionals, and medical students, were excluded from the study. A unique identification number was automatically assigned to each recorded response.

Statistical analysis plan 

The data were analysed using IBM SPSS Statistics Version 30.0 (IBM Corp., Armonk, USA). Descriptive statistics were performed. Comparison of the level of comfort and confidence between different work experiences was done using the Kruskal-Wallis test, followed by post-hoc analysis. P-value <0.05 was considered statistically significant. 

Ethical considerations 

Written informed consent was obtained from all participants prior to response collection to the survey. No incentive was provided to the participants. This study posed no physical risk to participants. The names and any identifying details of the participants were not asked to protect the privacy of the participants. The data collected were password-protected, and all documents were stored in a password-protected folder to which only the researchers had access. 

Researcher reflexivity

This study was conducted by a team of three international medical graduates (IMGs), one male and two female clinicians, who are themselves relatively new to the NHS. Our personal experiences navigating UK-specific clinical expectations, communication practices, and unfamiliar administrative processes such as ReSPECT and death certification may have shaped both our interest in the topic and our interpretation of the findings. Being early-career IMGs may have heightened our awareness of the challenges related to confidence, training gaps, and senior support, potentially influencing the selection and framing of survey items. Although we undertook steps to minimise bias, such as using expert validation during questionnaire development and applying objective statistical analysis, we acknowledge that our positionality may have contributed to emphasising certain themes over others. Recognizing this reflexive standpoint enhances the transparency of the research process and acknowledges that our insights are informed, in part, by our lived experience of transitioning into a new healthcare system.

## Results

ReSPECT discussion 

This study included 109 participants, of whom 46 (42.2%) were UK graduates and 63 (57.8%) were IMGs. Most of the participants were FY1 doctors (10.1%), foundation year 2 (FY2) doctors (11.9%), trust grade FY2 doctors (10.1%), and senior house officers (SHO) or above (67.9%) working within the NHS. The majority of the participants had an experience of 1-3 years (47.7%). The majority of participants had not received any formal training in conducting ReSPECT discussions (74.3%). 

Although clinicians from all selected trusts were invited to participate, the response rates varied across sites. Approximately 60% of the total sample was contributed by two trusts, while the remaining trusts each provided smaller numbers of respondents. Recruiting from multiple hospitals and trusts enabled the inclusion of a broad mix of clinical roles, training backgrounds, and levels of experience, thereby capturing a wide spectrum of perspectives on ReSPECT discussions and death verification processes.

Most of the participants (33.9%) said that they had participated 1-5 times in ReSPECT discussions in the last six months, and time constraint (48.6%) and fear of causing distress (42.2%) were the most common challenges faced by them during ReSPECT discussions. According to the majority of participants (78.9%), experience and exposure play an important role in increasing their confidence in having these discussions. They said that observation of senior colleagues (50.5%), workshop/seminar (45.9%), and simulation training (44.0%) were the most effective and helpful training/support methods (Table 1). 

**Table 1:** Response of participants to each question Items relating to the ReSPECT process were based on the Recommended Summary Plan for Emergency Care and Treatment (ReSPECT) framework developed by the Resuscitation Council UK [4,5].

Question	Response	Number of participants (N=109)
ReSPECT discussion		
How many years of clinical experience do you have?	Less than 1 year	14 (12.8%)
	1-3 years	52 (47.7%)
	4-6 years	17 (15.6%)
	More than 6 years	26 (23.9%)
Have you received formal training in conducting ReSPECT discussion?	Yes	28 (25.7%)
	No	81 (74.3%)
How often have you participated in ReSPECT discussion in last 6 months?	Never	17 (15.6%)
	1-5 times	37 (33.9%)
	5-10 times	24 (22.0%)
	More than 10 times	31 (28.4%)
What are the main challenges you face during ReSPECT discussion?	Lack of confidence	19 (17.4%)
	Emotional difficulty	27 (24.8%)
	Lack of experience	32 (29.4%)
	Fear of causing distress	46 (42.2%)
	Language or cultural barriers	25 (22.9%)
	Lack of senior support	21 (19.3%)
	Time constraints	53 (48.6%)
What helps increase your confidence in having these discussions?	Observing senior staff	59 (54.1%)
	Formal training	55 (50.5%)
	Clear guidelines and protocol	42 (38.5%)
	Experience/exposure	86 (78.9%)
	Supportive team environment	49 (45.0%)
What type of training/support would you find most helpful?	Simulation training	48 (44.0%)
	Role play	43 (39.4%)
	Workshops/seminars	50 (45.9%)
	Observation of senior colleagues	55 (50.5%)
	E-learning modules	25 (22.9%)
	Peer discussion/debrief	43 (39.4%)
Death certification		
Have you received formal training in completing death verification forms and preparing death certificates accurately?	Yes	43 (39.4%)
	No	66 (60.6%)
How often have you completed death verification form and death certificates in last 6 months?	Never	30 (27.5%)
	1-5 times	57 (52.3%)
	5-10 times	14 (12.8%)
	More than 10 times	8 (7.3%)
What are the main challenges you face during completing death verification forms and preparing death certificates accurately?	Lack of confidence	19 (17.4%)
	Emotional difficulty	15 (13.8%)
	Lack of experience	37 (33.9%)
	Fear of causing distress	24 (22.0%)
	Language or cultural barriers	15 (13.8%)
	Lack of senior support	27 (24.8%)
	Time constraints	44 (40.4%)
What helps increase your confidence in completing death verification forms and preparing death certificates accurately?	Observing senior staff	33 (30.3%)
	Formal training	58 (53.2%)
	Clear guidelines and protocol	62 (56.9%)
	Experience/exposure	54 (49.5%)
	Supportive team environment	36 (33.0%)
What type of training/support would you find most helpful?	Simulation training	42 (38.5%)
	Workshops/seminars	61 (56.0%)
	Observation of senior colleagues	41 (37.6%)
	E-learning modules	42 (38.5%)
	Peer discussion/debrief	36 (33.0%)

Death certification 

On asking about the death certificate, 66 (60.6%) participants said that they had not received any formal training for the same. Most of the participants (52.3%) had completed death certificate forms 1-5 times in the last six months. Time constraint (40.4%), lack of experience (33.9%), and lack of senior support (24.8%) were the most common challenges faced during death verification forms and death certificate preparation. According to most of the participants, clear guidelines and protocol (56.9%) and formal training (53.2%) would be helpful in increasing their confidence in completing death verification forms and preparing death certificates accurately. Most of the participants (56.0%) found workshops/seminars to be the most effective type of training/support pertaining to death verification and certificate forms (Table 1). 

Confidence level 

The confidence level of participants with respect to ReSPECT and death certification was scored on a scale of 1 to 5. The median (interquartile range) score for confidence in both domains is presented in Table 2. 

**Table 2:** Description of level of confidence among study participants ReSPECT-related confidence questions were formulated based on the Resuscitation Council UK’s Recommended Summary Plan for Emergency Care and Treatment (ReSPECT) [4,5]. IQR: Interquartile range

Domain	Question	Score					Median (IQR) score
		Score 1	Score 2	Score 3	Score 4	Score 5	
ReSPECT	How confident do you feel initiating a ReSPECT discussion with a patient or their family?	10 (9.2%)	10 (9.2%)	21 (19.3%)	43 (39.4%)	25 (22.9%)	4.0 (3.0-4.0)
	How confident are you in explaining the purpose of the ReSPECT form?	8 (7.3%)	8 (7.3%)	19 (17.4%)	47 (43.1%)	27 (24.8%)	4.0 (3.0-4.5)
	How confident do you feel in explaining the end-of-life care preferences with families?	5 (4.6%)	13 (11.9%)	28 (25.7%)	39 (35.8%)	24 (22.0%)	4.0 (3.0-4.0)
	How confident do you feel in managing emotional responses from families during the discussion?	6 (5.5%)	10 (9.2%)	30 (27.5%)	42 (38.5%)	21 (19.3%)	4.0 (3.0-4.0)
	How confident do you feel in documenting the outcome of the ReSPECT discussion accurately?	5 (4.6%)	11 (10.1%)	23 (21.1%)	45 (41.3%)	25 (22.9%)	4.0 (3.0-4.0)
	How confident are you in discussing DNACPR (Do Not Attempt CPR) within the ReSPECT conversation?	5 (4.6%)	5 (4.6%)	19 (17.4%)	49 (45.0%)	31 (28.4%)	4.0 (3.0-5.0)
Death	How comfortable are you with completing death verification forms and preparing death certificates accurately?	7 (6.4%)	14 (12.8%)	23 (21.1%)	43 (39.4%)	22 (20.2%)	4.0 (3.0-4.0)
	How confident do you feel in managing emotional responses from families during the discussion?	6 (5.5%)	9 (8.3%)	32 (29.4%)	41 (37.6%)	21 (19.3%)	4.0 (3.0-4.0)

On comparing the confidence between participants with different levels of experience, it was found that the confidence to initiate a ReSPECT discussion, to explain the purpose of the ReSPECT form, to explain the end-of-life care preferences with families, and to discuss Do Not Attempt CPR (DNACPR) was significantly greater among participants with experience of 1-3 years, 4-6 years and >6 years compared to participants with an experience of <1 year (p-value <0.05). 

The confidence to manage emotional responses from families during the discussion, to document the outcome of the ReSPECT discussion accurately, the level of comfort to complete death verification forms and prepare death certificates accurately, and confidence to manage emotional responses from families during the discussion did not differ significantly between participants with different levels of experience (p-value >0.05) (Table 3). 

**Table 3:** Comparison of level of confidence among participants with different levels of experience ReSPECT-related confidence questions were formulated based on the Resuscitation Council UK’s Recommended Summary Plan for Emergency Care and Treatment (ReSPECT) [4,5]. *indicates statistical significance. IQR: Interquartile range

ReSPECT												
Question	Median (IQR) score for confidence								Kruskal-Wallis H value	p-value	ε²	Effect size
	Less than 1 year (n=14)		1-3 years (n=52)		4-6 years (n=17)		>6 years (n=26)					
	Median (IQR)	Mean rank	Median (IQR)	Mean rank	Median (IQR)	Mean rank	Median (IQR)	Mean rank				
How confident do you feel initiating a ReSPECT discussion with a patient or their family?	3.0 (1.0-4.0)	36.39	3.5 (3.0-4.0)	47.99	4.0 (3.5-5.0)	68.56	4.0 (4.0-5.0)	70.17	18.005	<0.001*	0.143	Large
How confident are you in explaining the purpose of the ReSPECT form?	4.0 (1.75-4.0)	40.96	4.0 (3.0-4.0)	48.19	4.0 (4.0-5.0)	69.56	4.0 (4.0-5.0)	66.65	13.702	0.003*	0.102	Moderate
How confident do you feel in explaining the end-of-life care preferences with families?	3.0 (2.0-4.0)	34.25	3.0 (3.0-4.0)	45.45	4.0 (4.0-5.0)	67.50	4.5 (4.0-5.0)	77.10	28.265	<0.001*	0.240	Large
How confident do you feel in managing emotional responses from families during the discussion?	4.0 (2.0-4.25)	50.54	3.0 (3.0-4.0)	49.13	4.0 (4.0-4.5)	63.82	4.0 (3.0-5.0)	63.37	5.706	0.127	0.026	Small
How confident do you feel in documenting the outcome of the ReSPECT discussion accurately?	4.0 (2.0-4.25)	48.93	4.0 (3.0-4.0)	48.96	4.0 (3.0-5.0)	63.29	4.0 (4.0-5.0)	64.92	6.776	0.079	0.036	Small
How confident are you in discussing DNACPR (Do Not Attempt CPR) within the ReSPECT conversation?	4.0 (2.75-4.25)	49.57	4.0 (3.0-4.0)	47.71	4.0 (3.5-5.0)	70.82	4.0 (4.0-5.0)	62.15	9.957	0.019*	0.066	Moderate
Death certification												
How comfortable are you with completing death verification forms and preparing death certificates accurately?	4.0 (2.75-4.25)	53.36	3.0 (2.25-4.0)	48.41	4.0 (3.5-5.0)	65.62	4.0 (4.0-4.0)	62.12	6.021	0.111	0.029	Small
How confident do you feel in managing emotional responses from families during the discussion?	3.5 (2.0-4.0)	45.04	4.0 (3.0-4.0)	52.93	4.0 (3.0-5.0)	64.85	4.0 (3.0-4.0)	58.06	3.840	0.279	0.008	Negligible

## Discussion

This survey underscores a persistent gap between the ethical and legal responsibilities that NHS clinicians face at the end of life and the formal training provided to prepare them. Despite the widespread implementation of the ReSPECT across the UK, only a minority of respondents reported any structured education in this process. Physicians and medical students frequently report feeling inadequately prepared or uneasy when discussing emergency care, treatment options, or end-of-life issues with patients and their families [15]. Christakis and Iwashyna stated that inadequate training for communicating prognosis is one of the reasons why clinicians avoid initiating end-of-life discussions [16]. Initiating timely and transparent discussions with patients about their diagnoses and advance care planning enables both patients and their families to make informed decisions about future medical treatment. Such conversations help reduce anxiety and suffering while supporting patients in achieving a dignified and peaceful end-of-life experience [15].

In this study, across both domains, three outcomes emerged consistently: (i) Deficit in formal training: Most participants had not received structured teaching on either ReSPECT conversations or death certification; (ii) Low confidence: Particularly among IMGs and early-career doctors, confidence levels were strikingly low, with many respondents rating themselves at the lowest end of the scale; (iii) Need for structured support: Respondents repeatedly emphasized the need for clear guidelines, structured observation, simulation training, and supportive senior mentorship to navigate these ethically challenging areas.

In the present study, time constraint (48.6%) and fear of causing distress (42.2%) were the most common challenges faced by clinicians during ReSPECT discussions. In the study by Perkins et al., clinicians reported that lack of time prevented more conversations [5]. Time constraints in the acute hospital setting have been identified as a cause for inconclusive or partial outcomes by Eli et al. [17]. In another study, Eli et al. found that planned conversations were frequently hindered by timing challenges and time limitations, causing physicians to postpone such discussions, sometimes indefinitely [18]. Previous literature revealed that participants described fears of “saying the wrong thing” as a recurring barrier in implementing ReSPECT. Similar anxieties have been identified in qualitative research on resuscitation discussions, where clinicians often experience moral distress and burnout when navigating these emotionally charged situations without adequate support [12,19].

This study found that confidence was notably low among participants, mirroring earlier UK and international studies that found end-of-life communication skills to be insufficiently addressed during undergraduate and foundation training [5,6]. It was also found that confidence to initiate a ReSPECT discussion, explain the purpose of the ReSPECT form, discuss end-of-life care preferences with families, document the outcomes accurately, and address DNACPR decisions increased with experience and exposure (p<0.05).

In this study, the majority of participants said that observing senior colleagues (50.5%), workshops/seminars (45.9%), and simulation training (44.0%) were the most effective and helpful methods for improving confidence in the ReSPECT process. The fact that confidence improved with exposure and senior mentorship suggests that these skills are best developed through experiential learning rather than didactic instruction alone. Simulation-based training and supervised clinical encounters have previously been shown to enhance clinicians’ communication confidence and reduce stress when discussing advanced care planning [10].

Death verification and certification presented parallel challenges. Many respondents reported little to no formal training in completing statutory forms or understanding legal requirements, echoing prior studies documenting frequent administrative errors and delays in death certification [11]. In our study, many respondents reported no formal training (60.0%) in completing statutory forms or understanding legal requirements, again aligning with prior findings [11]. Participants consistently expressed time constraints (40.4%), lack of experience (33.9%), lack of confidence (17.4%), emotional difficulty (13.8%), language or cultural barriers (13.8%), lack of senior support (24.8%), and fear of causing distress (22.0%). Participants said that workshops/seminars (56.0%) could be the most effective type of training/support for death verification and certification. The usefulness of workshops has been reported in a study conducted by Lakkireddy et al., where only 15.5% of participants were initially able to correctly determine the cause of death. However, after attending a workshop or reviewing printed guidelines, the accuracy rate significantly improved to 84.5% [20].

Research indicates that deficiencies in death certification can have downstream effects on public health data accuracy and bereaved families’ experiences, highlighting the importance of targeted education in this domain [21]. Embedding ReSPECT and death certification education within mandatory training, appraisal processes, and postgraduate curricula would signal institutional commitment and help normalise these essential conversations as a core part of clinical professionalism [6,22].

Limitations of the study

Convenience sampling was used to recruit the requisite number of participants from hospitals across multiple NHS trusts in the UK. This method allowed for timely and practical data collection; however, it also meant that participation depended on clinicians’ availability and willingness to respond. As a result, the sample may not fully represent all clinicians working across the NHS, particularly those in busier or more specialized settings, and this may limit the generalizability of the findings. As a result, the conclusions drawn may not accurately reflect the broader population of interest.

Although participants were recruited from 10 hospitals across 10 NHS trusts, participation rates varied considerably between sites. Notably, approximately 60% of the total sample (109 participants) were drawn from only two trusts, while the remaining trusts contributed far fewer responses. This uneven distribution introduces the potential for differential participation bias, as the views, experiences, and training exposure of clinicians from more highly represented trusts may disproportionately influence the overall findings. Trust-level variations in education, ReSPECT implementation, documentation practices, and availability of senior support may therefore be under- or over-represented in the dataset. As a result, the sample may not fully reflect the diversity of clinical environments across the NHS, and the generalizability of the findings to all trusts must be interpreted with caution.

Thirdly, the study relied on self-reported data, which is susceptible to social desirability bias. Given the ethical and professional expectations surrounding ReSPECT discussions and death verification, participants may have felt pressure to present themselves as more confident, knowledgeable, or prepared than they truly were. Clinicians may have been hesitant to admit gaps in training or low confidence for fear of appearing inadequate or professionally inexperienced. This could lead to overestimation of confidence levels and underreporting of challenges or training needs, thereby influencing the interpretation of key variables.

Another consideration is the potential for response bias, as participation in the survey was voluntary. Clinicians who chose to respond may differ systematically from those who did not, for example, individuals with a stronger interest in ReSPECT, greater concerns about training, or more available time may have been more likely to participate. This could lead to overrepresentation of certain viewpoints and underrepresentation of others, particularly from clinicians with heavier workloads or lower confidence, whose perspectives may be equally important but less frequently captured.

Although the Kruskal-Wallis test was used to compare confidence levels across experience groups, effect sizes (such as eta-squared) were not calculated. As a result, the magnitude of observed differences could not be quantified beyond statistical significance.

Next, recall bias may have affected participants' reports of the frequency of ReSPECT discussions or death certification tasks, particularly if these occurred months prior to the survey. Finally, because the study was conducted by early-career IMGs, researchers' reflexivity must be acknowledged. Their own experiences navigating NHS systems and training structures may have influenced the framing of the questionnaire and the emphasis placed on specific findings, despite efforts to minimize bias through expert validation and structured analysis.

Recommendations for future research

Future studies may consider recruiting a larger sample using probability-based sampling methods to enhance representativeness and improve the generalizability of findings. A stratified sampling process across trusts and specialties, repeated reminders to encourage broader participation, and monitoring response rates in real time to identify underrepresented groups can address non-response bias. Larger, more diverse samples across multiple NHS trusts would allow for meaningful subgroup analyses and reduce bias, for example, comparing UK graduates with IMGs or junior doctors with senior clinicians. Such comparisons could provide deeper insights into the variability of experiences or outcomes among distinct demographic or professional groups. A more robust sampling technique would help mitigate sampling bias and strengthen external validity.

Incorporating a mixed-methods design that includes qualitative components such as focus group discussions or in-depth interviews could offer richer, more nuanced insights while also addressing the effects of social desirability bias. Anonymous qualitative interviews or focus groups may allow clinicians to more openly explore their uncertainties, emotional challenges, and training needs without the perceived judgment associated with self-rating confidence. Including objective measures such as audits of death certification accuracy or assessments within simulation-based training could complement self-reported data and reduce bias. Qualitative data would allow in-depth exploration of underlying themes, contextual factors, and participant perspectives that may not be fully captured through quantitative measures alone. This approach could enhance the overall understanding of the complex ethical issues and support the development of more targeted interventions or recommendations.

Further longitudinal research would also be valuable to examine how confidence and competence evolve over time, particularly as clinicians progress through training or receive structured educational interventions. Finally, future studies should further investigate the experiences of IMGs, who may face unique barriers related to communication, cultural expectations, and familiarity with UK-specific legal and ethical processes.

## Conclusions

This study highlights important gaps in confidence and formal training related to the ReSPECT process and death verification among a sample of NHS clinicians. A substantial proportion of participants reported limited structured training in both domains, with lower confidence particularly evident among early-career doctors and those with less clinical experience. While experience and exposure were associated with higher confidence in several ReSPECT-related tasks, challenges such as time constraints, fear of causing distress, and limited senior support remained common across experience levels.

Given the cross-sectional design, convenience sampling, and relatively small sample size, these findings should be interpreted as exploratory rather than representative of all NHS clinicians. Nevertheless, the results suggest that targeted, practice-based educational approaches such as workshops, simulation training, observation of senior clinicians, and clear institutional guidance may help address identified confidence gaps. Further research using larger, more representative samples and longitudinal designs is needed to confirm these findings and to evaluate the impact of structured training interventions on clinician confidence and competence in end-of-life communication and death certification.

## Appendices

Questionnaire

Note: The questionnaire was developed by the authors of this study.

Ethical Challenges in Clinical Practice: A Questionnaire on ReSPECT Discussions and Death Verification Procedures

*Indicates required question

Background

1. Graduation * Mark only one oval. UK IMG

2. Role in NHS * Mark only one oval. F1 F2 Trust grade F2 SHO and above 3. How many years of clinical experience do you have? Mark only one oval. Less than 1 year 1 - 3 years 4 - 6 years More than 6 years

ReSPECT (Resuscitation plan) Discussion

4. Have you received formal training in conducting ReSPECT discussion? * Mark only one oval. Yes No

5. How often have you participated in ReSPECT discussion in last 6 months? * Mark only one oval. Never 1 - 5 times 5 - 10 times More than 10 times

6. How confident do you feel initiating a ReSPECT Discussion with a patient or their family? * Mark only one oval. Least    1    2    3    4    5    Very Confident 7. How confident are you in explaining the purpose of the ReSPECT form? * Mark only one oval. Least    1    2    3    4    5    Very Confident

8. How confident do you feel in explaining the end-of-life care preferences with families? * Mark only one oval. Least    1    2    3    4    5    Very Confident

9. How confident do you feel in managing emotional responses from families during the discussion? * Mark only one oval. Least    1    2    3    4    5    Very Confident

10. How confident do you feel in documenting the outcome of the ReSPECT discussion accurately? * Mark only one oval. Least    1    2    3    4    5    Very Confident 11. How confident are you in discussing DNACPR (Do Not Attempt CPR) within the ReSPECT conversation? * Mark only one oval. Least    1    2    3    4    5    Very Confident

12. What are the main challenges you face during ReSPECT discussion? * Tick all that apply. Lack of confidence Emotional difficulty Lack of experience Fear of causing distress Language or cultural barriers Lack of senior support Time constraints

13. What helps increase your confidence in having these discussions? * Tick all that apply. Observing senior staff Formal training Clear guidelines and protocol Experience/exposure Supportive team environment 14. What type of training /support would you find most helpful? * Tick all that apply. Simulation training Role play Workshops/seminars Observation of senior colleagues E-learning modules Peer discussion/ debrief

Death Verification Form and Death Certificates 

15. Have you received formal training in completing death verification forms and preparing death certificates accurately? * Mark only one oval. Yes No

16. How often have you completed death verification form and death certificates in last 6 months? * Mark only one oval. Never 1 - 5 times 5 - 10 times More than 10 times 17. How comfortable are you with completing death verification forms and preparing death certificates accurately? * Mark only one oval. Least    1    2    3    4    5    Most Comfortable

18. How confident do you feel in managing emotional responses from families during the discussion? * Mark only one oval. Least    1    2    3    4    5    Very Confident

19. What are the main challenges you face during completing death verification forms and preparing death certificates accurately? * Tick all that apply. Lack of confidence Emotional difficulty Lack of experience Fear of causing distress Language or cultural barriers Lack of senior support Time constraints 20. What helps increase your confidence in completing death verification forms and preparing death certificates accurately? * Tick all that apply. Observing senior staff Formal training Clear guidelines and protocol Experience/exposure Supportive team environment

21. What type of training /support would you find most helpful? * Tick all that apply. Simulation training Workshops/seminars Observation of senior colleagues E-learning modules Peer discussion/debrief
